# Analysis of left-turn behaviors of non-motorized vehicles and vehicle-bicycle conflicts

**DOI:** 10.1371/journal.pone.0291504

**Published:** 2023-09-14

**Authors:** Tianjun Feng, Jingyao Liu, Chunyan Liang, Xiujuan Tian, Chun Chen, Keke Liu

**Affiliations:** School of Transportation Science and Engineering, Jilin Jianzhu University, Changchun, Jilin, China; University of Shanghai for Science and Technology, CHINA

## Abstract

In order to further study the expansion characteristics of left-turning non-motorized vehicles at intersections and the relationship between expansion characteristics and vehicle-bicycle conflicts, the trajectory point data of left-turning non-motorized vehicles are extracted using video trajectory tracking technology, and construct the cubic curve expansion envelope equation with the highest fitting degree. For the purpose of quantifying the expansion degree of non-motor vehicles after starting, two intersections in Guangxi Zhuang Autonomous Region were selected for case analysis, and the numerical range of expansion degree of the intersection with a left-turn waiting area and the intersection without a left-turn waiting area was obtained. Study the mathematical relationship between the expansion degree and its influencing factors, and establish the multivariate nonlinear regression equation between the expansion degree and the left-turn non-motorized vehicle flow, the number of parallel non-motorized vehicles, and the left-turn green light time. Analyze the vehicle-bicycle conflicts caused by the expansion of left-turning non-motorized vehicles, determine the essential factors affecting the number of non-motorized vehicles, and establish the multiple linear regression equation between the number of non-motorized vehicles and the number of left-turning non-motorized vehicles, the expansion degree, and the number of parallel non-motorized vehicles, the results show that the model has high accuracy. By analyzing the expansion characteristics of left-turning non-motorized vehicles at intersections, the relationship between different influencing factors and the expansion degree is obtained. Then the vehicle-bicycle conflicts under the influence of expansion characteristics is analyzed, providing theoretical ideas for improving traffic efficiency and optimizing traffic organization at intersections.

## 1.Introduction

With the rapid development of global urbanization, the number of cars continues to increase, and the riding space of non-motor vehicles is squeezed and compressed. The mixed traffic composed of non-motor vehicles, motor vehicles, and pedestrians interferes with each other on the road, which has become an urban traffic problem. One of the roots of [1, 2]. As a green transportation mode, bicycle transportation has the characteristics of punctuality, travel flexibility, environmental protection, and health. It has become an excellent supplement to developing urban public transport systems and relieves road traffic pressure [3–5].

The intersection is the node of the urban road network, and the vehicle-bicycle conflicts at the intersection has become a hidden danger point for road traffic safety. In order to solve the problem of vehicle-bicycle conflictsat intersections and further improve traffic efficiency, scholars have researched the problem of vehicle-bicycle conflicts within intersections. Scholars mainly conduct research on non-motor vehicle traffic characteristics, mechanism research, vehicle-bicycle conflicts discrimination and prediction, safety impact assessment, andvehicle-bicycle conflicts technology application [6–14]. Shladover [15] et al. used video detection technology to capture the trajectory of bicycles to obtain the time distribution characteristics of bicycles crossing intersections. Jing Chunguang [16] conducted an in-depth study on the vehicle-bicycle conflictsof intersections and established a calculation model for the critical value of left-turn bicycle flow in traffic conflicts for two-phase intersections. In order to assess the safety conflict between motor vehicles and bicycles, Puscar [17] et al. used a computer vision system for safety diagnosis. In the case of different intersection layouts and right-of-way, the yield rate of bicycles may change significantly. Guo Y [18] et al. used the automatic traffic conflict technology to evaluate the safety of the unconventional left-turn outer lane, and the conflict of the approach road at the intersection with the outer left-turn lane was more serious.

The expansion characteristic of non-motorized vehicles is a particular attribute, especially when turning left and crossing the street. Research has shown that this characteristic is critical to keeping the vehicle running smoothly and operating efficiently. Therefore, in-depth research on the expansion characteristics of non-motor vehicles is of great significance for improving the release efficiency and safety of intersections. At present, many scholars have carried out research on the expansion characteristics of non-motorized vehicles. Among them, the expansion phenomenon of bicycle traffic flow is described by introducing the concept of expansion degree [19], which provides a theoretical basis for the traffic design of mixed-vehicle intersections. At the same time, the researchers also explored the interference impact of non-motor vehicle expansion on motor vehicles [20–22]. They quantitatively analyzed the impact of non-motor vehicles on the traffic capacity and operation delay of motor vehicles. In addition, the dynamic and static factors affecting the expansion characteristics of electric bicycles in the intersection vehicle-road environment are also analyzed, and the mathematical relationship between the influencing factors and the expansion degree is determined [23].

Scholars have done much research to extract vehicle trajectory data from traffic observation videos. By automatically tracking the trajectories of motor vehicles, non-motor vehicles, and pedestrians in the video and obtaining their real motion coordinates, this technology is useful for identifying traffic conflicts, evaluating the severity of conflicts, predicting traffic conflicts, and providing a theoretical basis for improving intersection management. It is of great significance to the analysis and evaluation of traffic safety [24–30].

At present, the research on the expansion characteristics of non-motor vehicles mainly focuses on the motion model and traffic flow model, the causes and influencing factors of expansion characteristics, and the relationship between traffic characteristics and expansion characteristics. However, the current academic research rarely discusses how to use the research results of non-motor vehicle expansion characteristics to carry out traffic safety assessments and establish the relationship between non-motor vehicle inflation characteristics and traffic conflicts. Therefore, this paper deeply studies the expansion characteristics of left-turning non-motor vehicles at intersections and their relationship with vehicle-bicycle conflicts. By analyzing the expansion characteristics of left-turning non-motor vehicles, the conflict risk between non-motor vehicles and motor vehicles is discussed. Based on these research results, we can better carry out traffic safety assessments and establish the relationship between the expansion characteristics of non-motor vehicles and traffic conflicts, which can provide suggestions for future traffic planning and management. In addition, this paper also innovatively puts forward the relationship model between left-turning non-motor vehicle flow, the number of parallel non-motor vehicles, the left-turning green time, and the expansion degree. It provides a scientific basis for intersection signal control and traffic planning.

This study takes the signalized intersection as the research object. Through field investigation and video analysis, the expansion characteristics of left-turning non-motor vehicles at the intersection are deeply studied considering whether there is a left-turn waiting area at the intersection, which provides theoretical ideas for improving the traffic efficiency at the intersection and optimizing the traffic organization. In addition, based on the changes in vehicle space-time trajectory data, the concept of expansion degree is introduced to quantify the expansion degree of non-motor vehicles after starting, which provides a theoretical basis for the traffic design of plane intersections. The mathematical relationship model of left-turning non-motor vehicle expansion degree and its influencing factors is established. Then, the important influencing factors of the number of vehicle-bicycle conflicts caused by the expansion characteristics of the left-turning non-motor vehicle at the intersection are determined, and the multiple linear regression model of the number of vehicle-bicycle conflicts at the intersection is established.

Future research can further explore the behavioral characteristics of non-motor vehicles, the behavioral characteristics of non-motor vehicle drivers, and the impact of the traffic environment on expansion characteristics. In urban traffic planning, it is necessary to consider the expansion characteristics of non-motor vehicles and determine the width and number of non-motor lanes, as well as the length and width of the left turn area at the intersection, to improve the traffic capacity of the road. In terms of traffic signal control, through traffic signal control and optimization, the left green time and non-motor vehicle flow can be reasonably allocated at intersections to reduce the impact of non-motor vehicle expansion characteristics on intersection traffic. In addition, the intelligent signal control system can automatically adjust the signal timing scheme according to the real-time traffic flow and the expansion characteristics of non-motor vehicles, to improve the traffic efficiency of the intersection. In terms of traffic management, differentiated management strategies can be proposed according to the differences of expansion characteristics of non-motor vehicles of different types and speeds, to improve the safety and efficiency of non-motor traffic. These research results can provide scientific basis and technical support for urban road design and traffic planning. Therefore, in future research, it is necessary to further explore the mechanism and law of the expansion characteristics of non-motor vehicles and their impact on traffic flow, to improve the scientificity and practicability of urban road design and traffic planning.

## 2.Materials and methods

### 2.1 Intersection coordinate system

**Fig 1:**
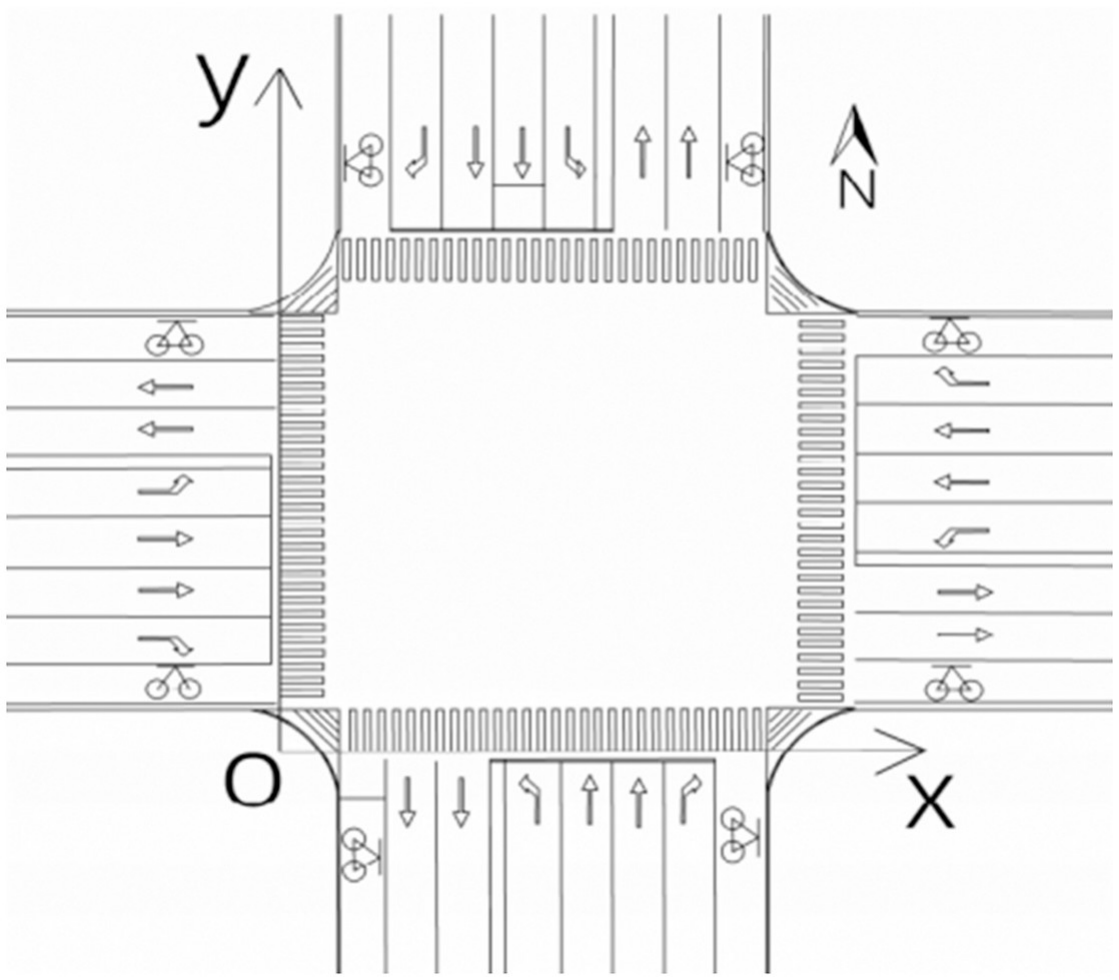
Intersection coordinate system.

For a typical cross-level intersection, the x-axis is the rear end line of the pedestrian crossing at the south entrance, and the y-axis is the rear end line of the pedestrian crossing at the west entrance. In this way, the two-dimensional coordinate system of the intersection is established, as shown in Fig 1. For irregular intersections, the corresponding coordinate system can be established by transforming the coordinates. The rear end line of one side of the crosswalk is selected as the x-axis, the apex of the rear end line is the origin, and the y-axis is established perpendicular to the x-axis direction through the origin, and the coordinates of the intersection are constructed.

### 2.2 Expansion envelope of left-turning non-motor vehicles

#### Data collection and processing

This paper selects two intersections of Luban Road-Daxuedong Road in Nanning City and Guizhong Avenue-Yingbin Road in Laibin City for data collection. Both intersections are typical four-phase intersections, and diversion islands are set up. There is a left-turn waiting area at the intersection of Luban Road-Daxuedong Road, but there is no left-turn waiting area at the intersection of Guizhong Avenue-Yingbin Road. The characteristic parameters of the intersection are shown in Table 1. UAV aerial photography obtains the traffic data during weekday evening peak hours of 17:30–19:30. The intersections are photographed for 3 hours, respectively, and a total of 102 cycles of samples are obtained. Using Tracker software to extract data from the collected video, The frame rate is 30.0 fps, and the measurement range in the video is set to the area surrounded by four crosswalks. Extract non-motor vehicle trajectory, flow, density, speed, and other data through video. Non-motor vehicle track point data is a collection of coordinate points of non-motor vehicles in the intersection coordinate system with the cycle as the time interval.

**Table 1:** Survey location characteristic parameters.

Intersection	Luban Road-Daxuedong Road		Guizhong Avenue-Yingbin Road	
*Direction*	East-West	North-South	Southwest—Northeast	Northwest—Southeast
*Numberoflanes*	12	10	8	8
Left Turn Bay	Left Turn Bay Installation		Absence of Left Turn Bay	
Form of separation between motor vehicles and non-motor vehicles	Marking isolation		Physical isolation	
*Numberofnon* − *motorizedlanes*	2	2	1	1
*Numberofleftturninletlanes*	2	2	2	2
*Typeofcentralseparator*	Green belt	guard bar	Green belt	guard bar
Phase green time(s)	Straight north-south direction 40s North-south left turn 50s Straight east-west direction 50s Left east west direction 43s		Southwest—Northwest turn left 37s Northwest—Southeast straight 54s Southeast—Southwest left 63s Southwest—Northeast straight 34s	
Cycle length (s)	195		200	

**Fig 2:**
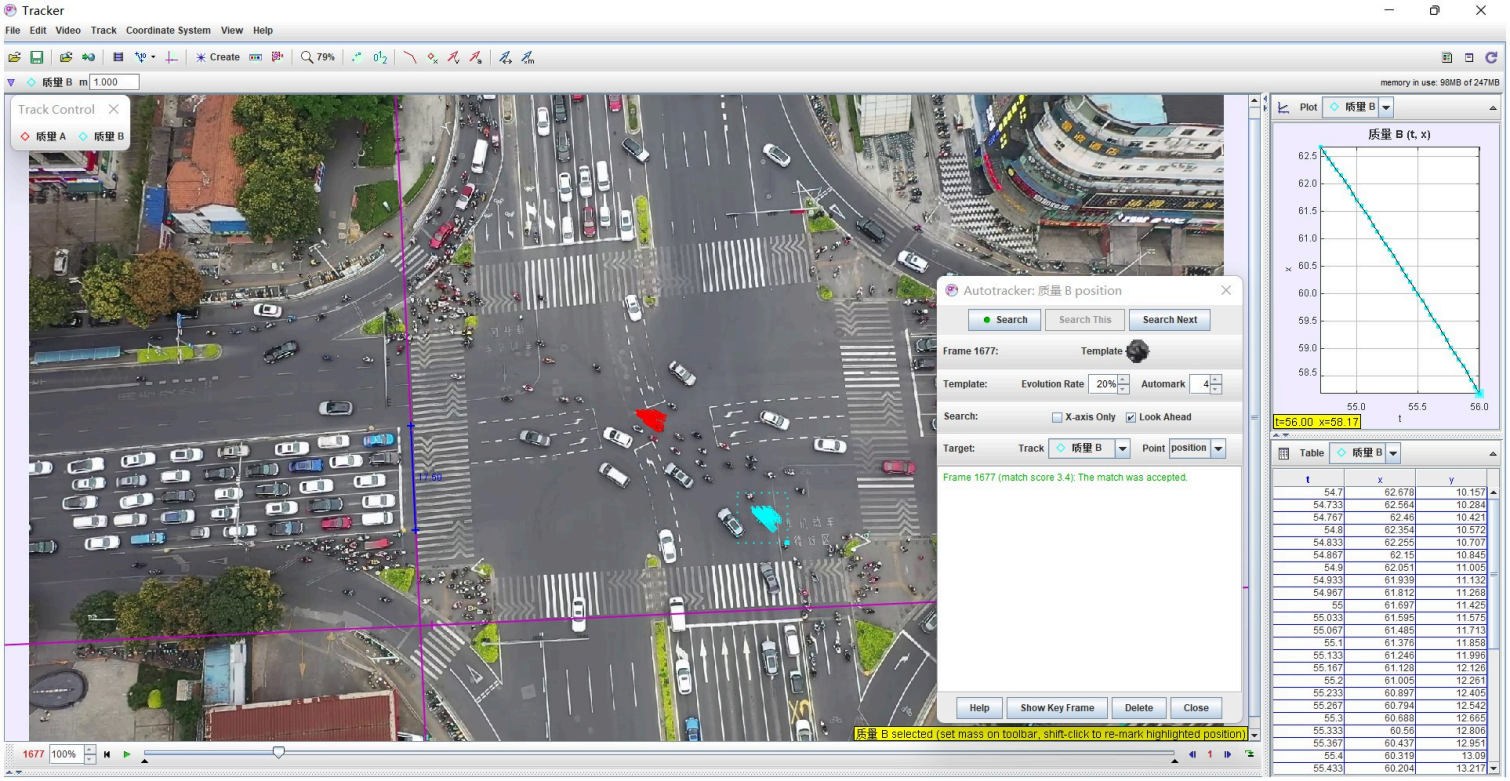
Left turn non motorized vehicle trajectory tracking.

The Tracker software is used to open the collected traffic video with a resolution of 1920 × 1080. First, the origin of the intersection coordinate system is determined, the direction of the x and y axes is determined, the two ends of the correction rod are pulled to both ends of a certain standard length in the video with the mouse, and the corresponding real length is input, to determine the spatial coordinate system of the video. Then set the start and end time bar of the video to complete the setting of the time coordinate system of the video. Finally, the tracking place is set according to the object to be tracked, the mouse selects the object to be tracked, and the automatic tracking mode is selected to track the tracked object. The automatic tracking mode will mark the location of each tracked object,as shown in Fig 2, and the corresponding position and value will also be synchronously marked in the graph and table generated by the software. Due to the small area and fast-moving speed of non-motor vehicles in the aerial video footage taken by drones, sometimes the automatic tracking software can not accurately track the situation, then the mouse is used to manually mark the position of each frame of the tracking object. After the final tracking is completed, the software can calculate the output chart and table, including the time of tracking the object, the horizontal coordinate point, and the vertical coordinate.

#### Data analysis of vehicle routing

**Fig 3:**
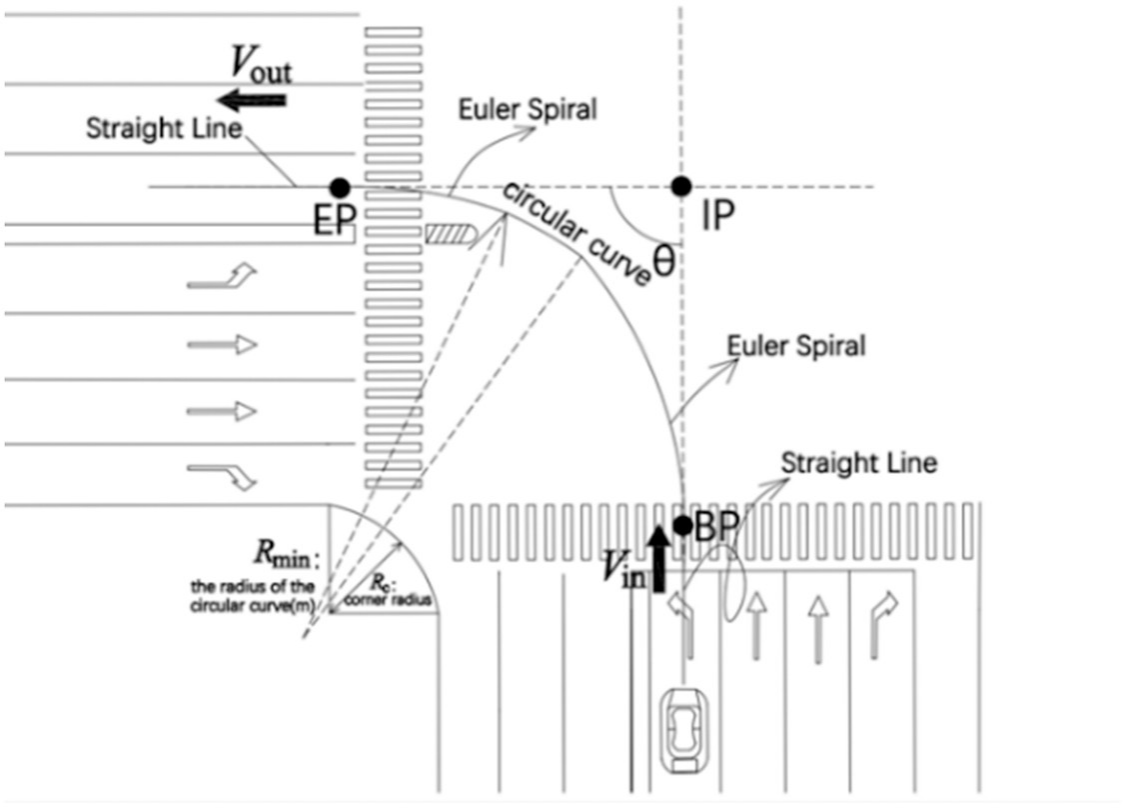
Trajectory approximation of left-turning vehicles.

According to the research of Alhajyaseen (2013), the path of a left-turning vehicle at a signalized intersection can be represented by a spline curve composed of 5 segments. This polygonal line comprises a straight line segment, an Euler spiral curve segment, and a circular curve segment. The path starts from the straight segment, then transitions to a linear Euler curve segment with a curvature of $1/A_{1}^{2}$., followed by a circular curve segment with a constant curvature of $1/R_{min}^{2}$, then an Euler curve segment with a linear curvature gradient of $-1/A_{2}^{2}$, and finally ends in a straight line segment. In order to uniquely determine this polygonal line, five parameters need to be defined: *A*_1_,*A*_2_, the starting point (BP) entering the Euler spiral curve and the end point (EP) exiting the Euler spiral curve, as shown in Fig 3.

**Fig 4:**
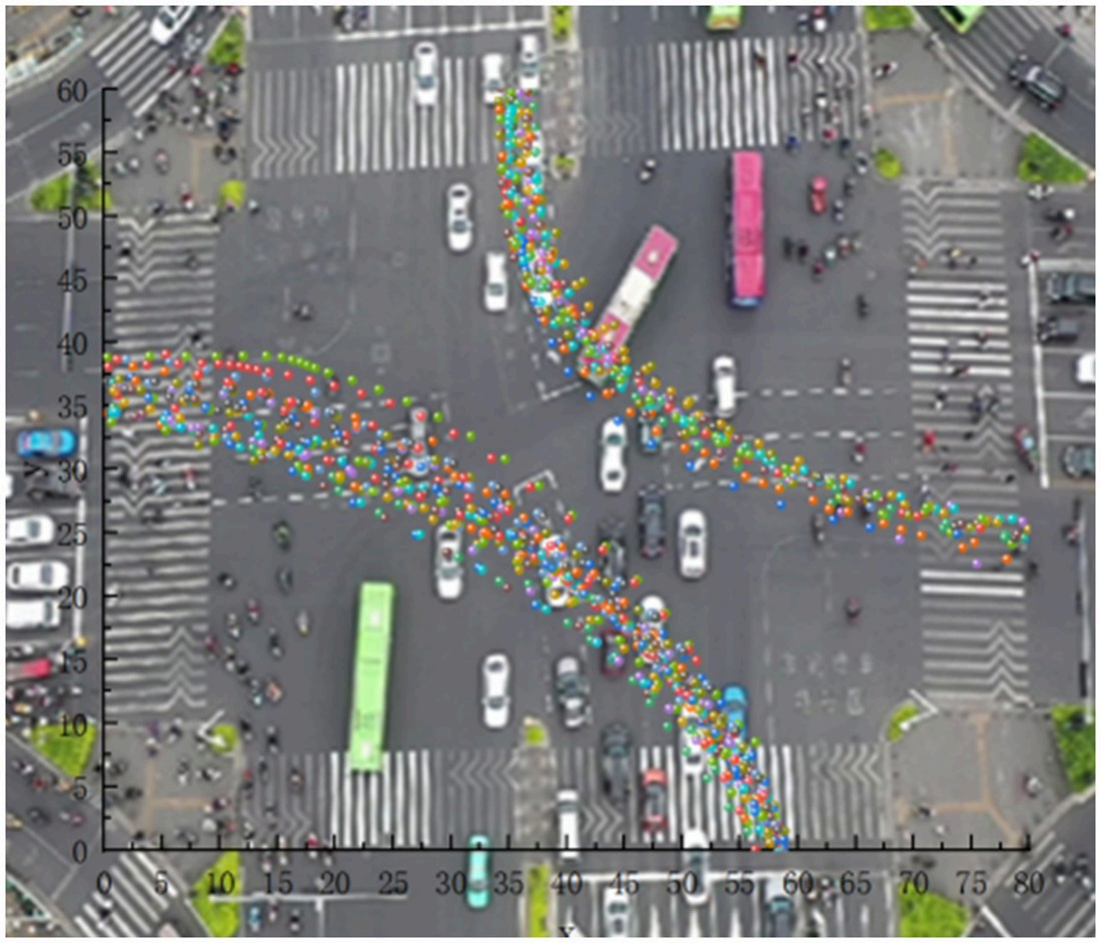
Left turning vehicle track diagram at Daxuedong Road—Luban Road intersection.

Through actual observation, the actual left-turn motor vehicle flow trajectory is found to conform to the assumed curve equation, as shown in Fig 4.

**Fig 5:**
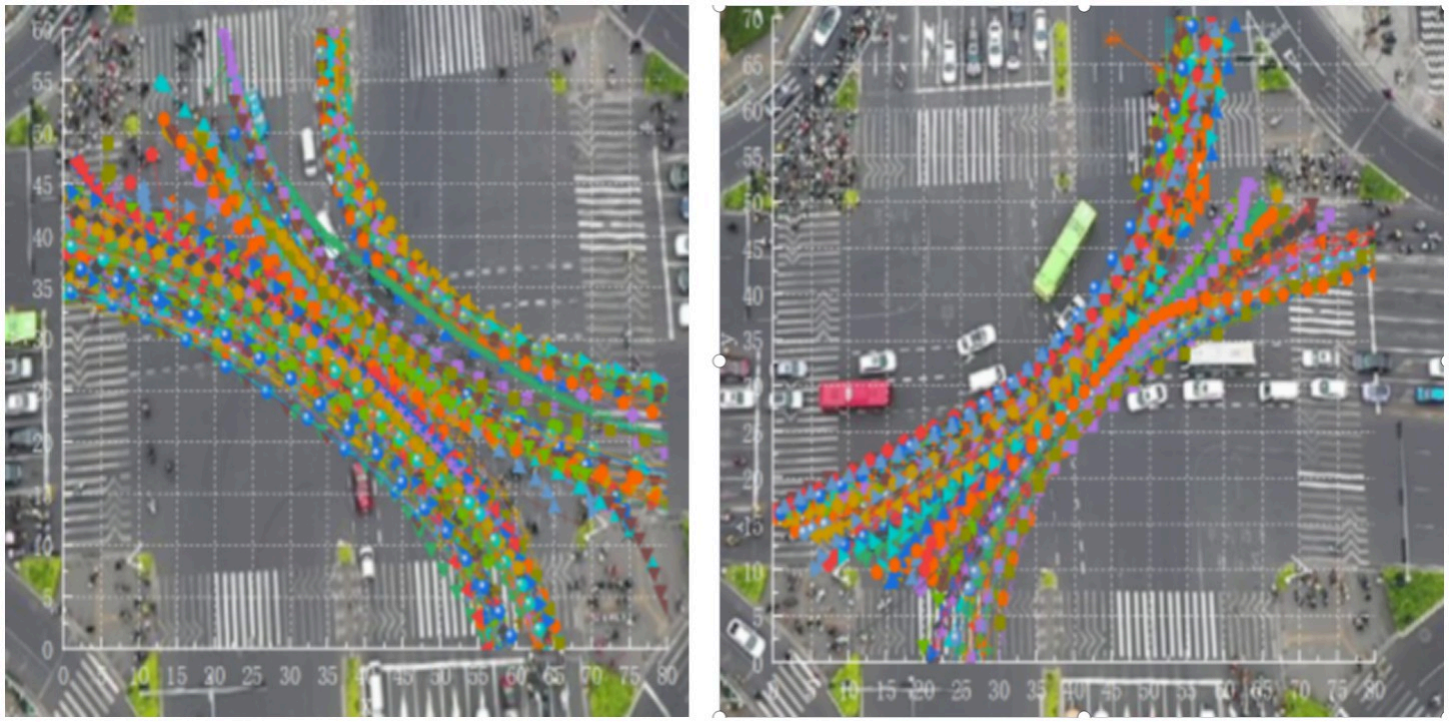
Left turn non motorized vehicles crossing trajectory at the intersection of Luban Road and Daxuedong Road.

**Fig 6:**
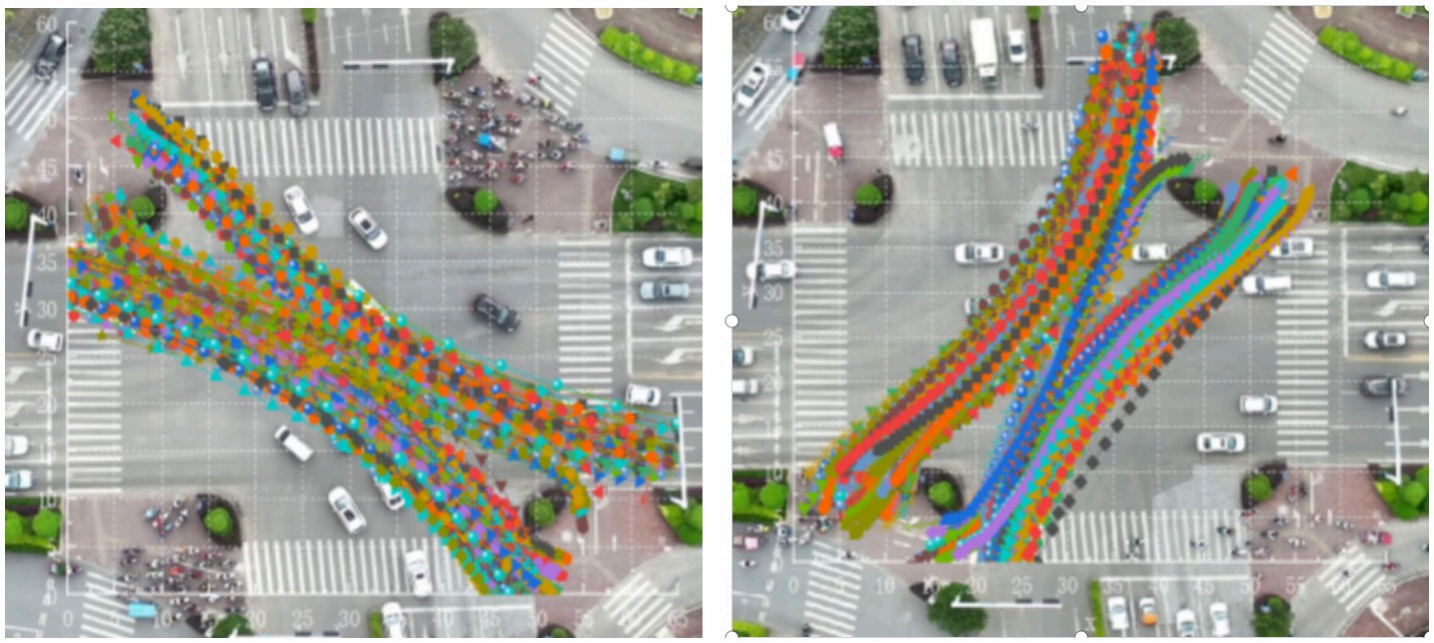
Left turn non motorized vehicles crossing trajectory at the intersection of Guizhong Avenue and Yingbin Road.

**Fig 7:**
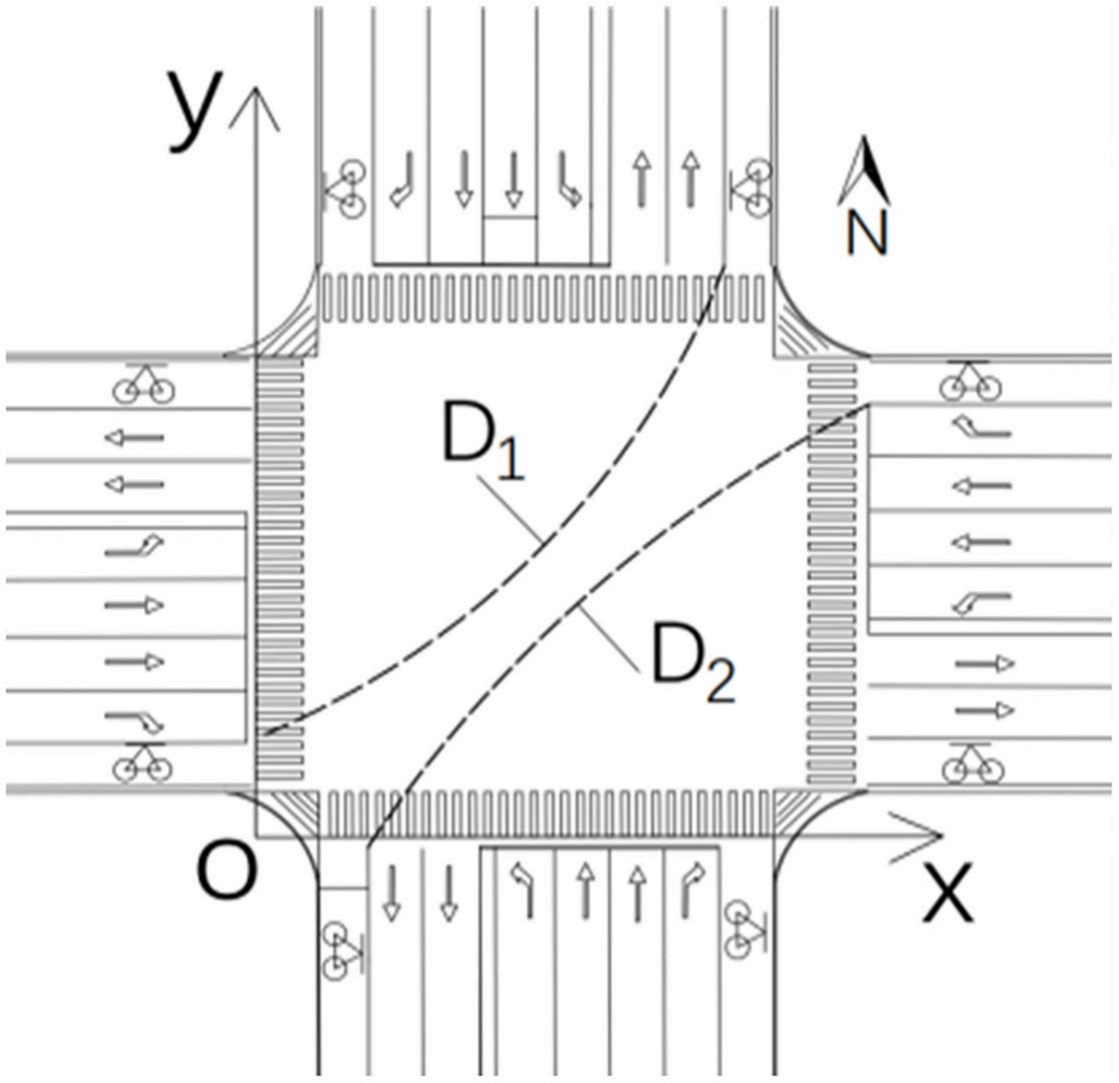
Expansion trajectory diagram of non motorized vehicles turning left at intersection.

When the green light is released at the intersection of Luban Road-Daxuedong Road, the flow of non-motor vehicles turning left expands and spreads to both sides, presenting an hourglass-shaped distribution, as shown in Fig 5. However, the two-way left-turning non-motor vehicle trajectories at the intersection of Guizhong Avenue-Yingbin Road respectively showed a tightening trend on both sides of the widest middle, as shown in Fig 6. Construct the expansion envelopes D1 and D2 of left-turn non-motorized vehicles, as shown in Fig 7.

At an intersection with a left turn waiting area, a motor vehicle may enter the intersection ahead of time and make a left turn in the area marked with a “Left turn waiting area” sign. When the flow of motor vehicles is large, the left turn waiting area is completely occupied by motor vehicles. When the green light starts, the left turn non-motor vehicles need to wait for the gap to pass through the left turn waiting area. Therefore, the street crossing track of the left turn non-motor vehicles is relatively more curved, and more attention needs to be paid to the driving situation of motor vehicles, so the speed and acceleration are relatively low. When there is no left turning zone for motor vehicles, the track of non-motor vehicles is relatively straight, and non-motor vehicles can complete the left turning operation more smoothly. So the velocity and acceleration are relatively high.

### 2.3 Curve fitting

**Fig 8:**
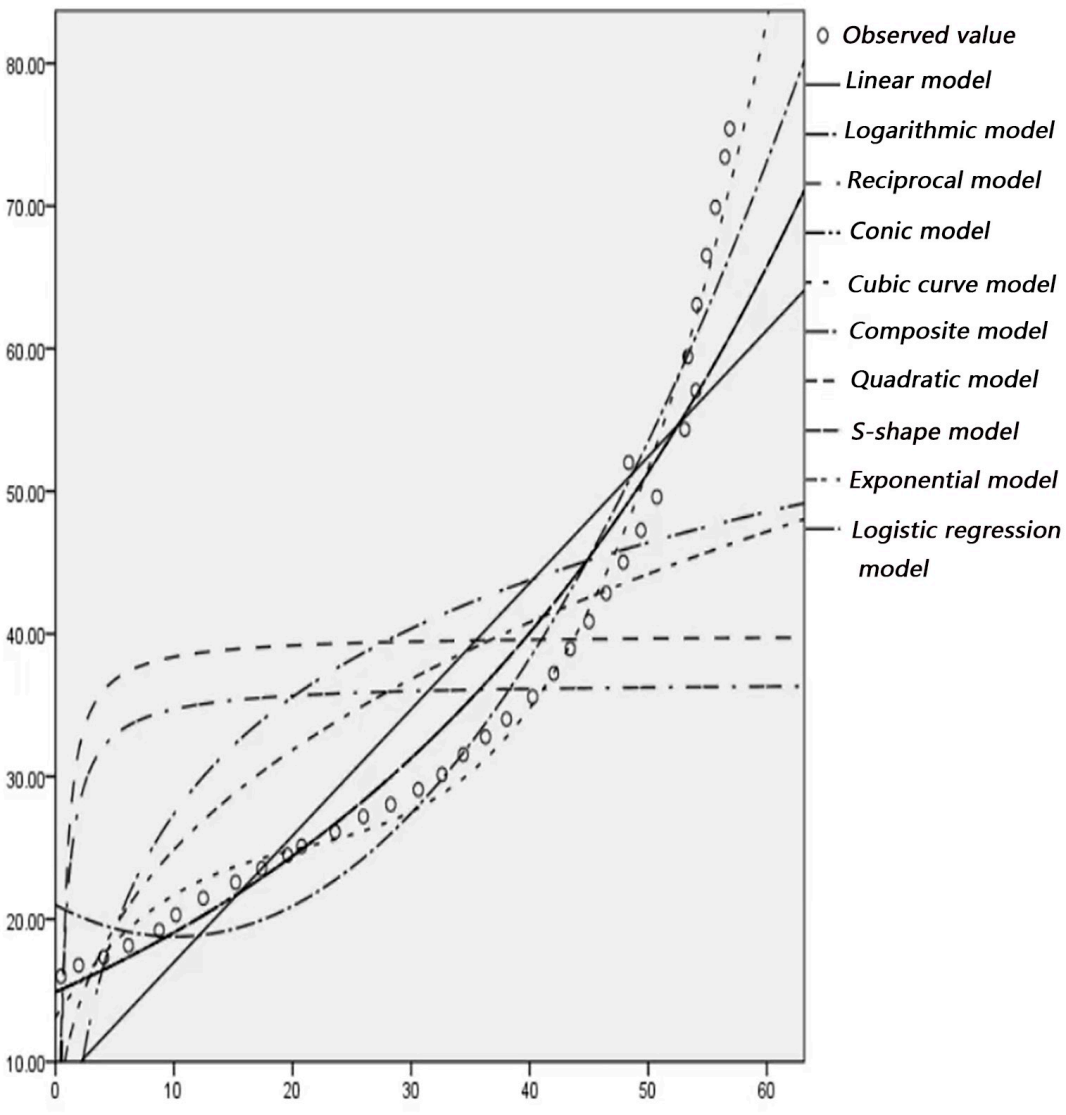
Curve fitting diagram of D1 at the intersection of Daxuedong Road and Luban Road.

**Fig 9:**
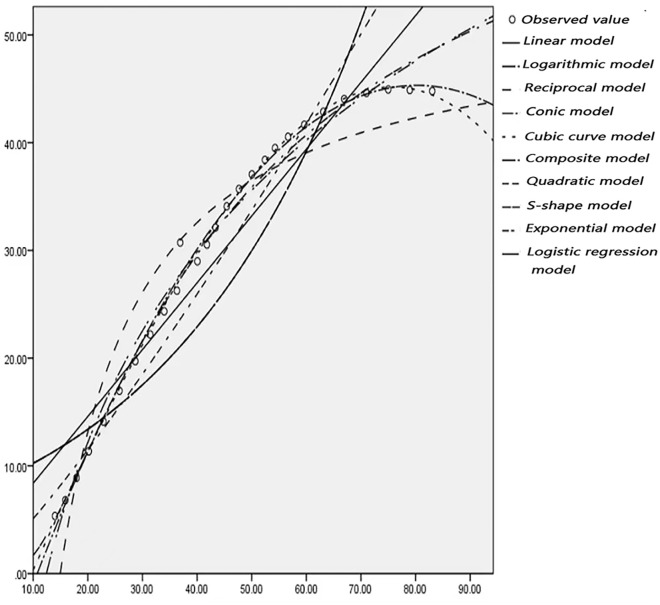
Curve fitting diagram of D2 at the intersection of Daxuedong Road and Luban Road.

**Fig 10:**
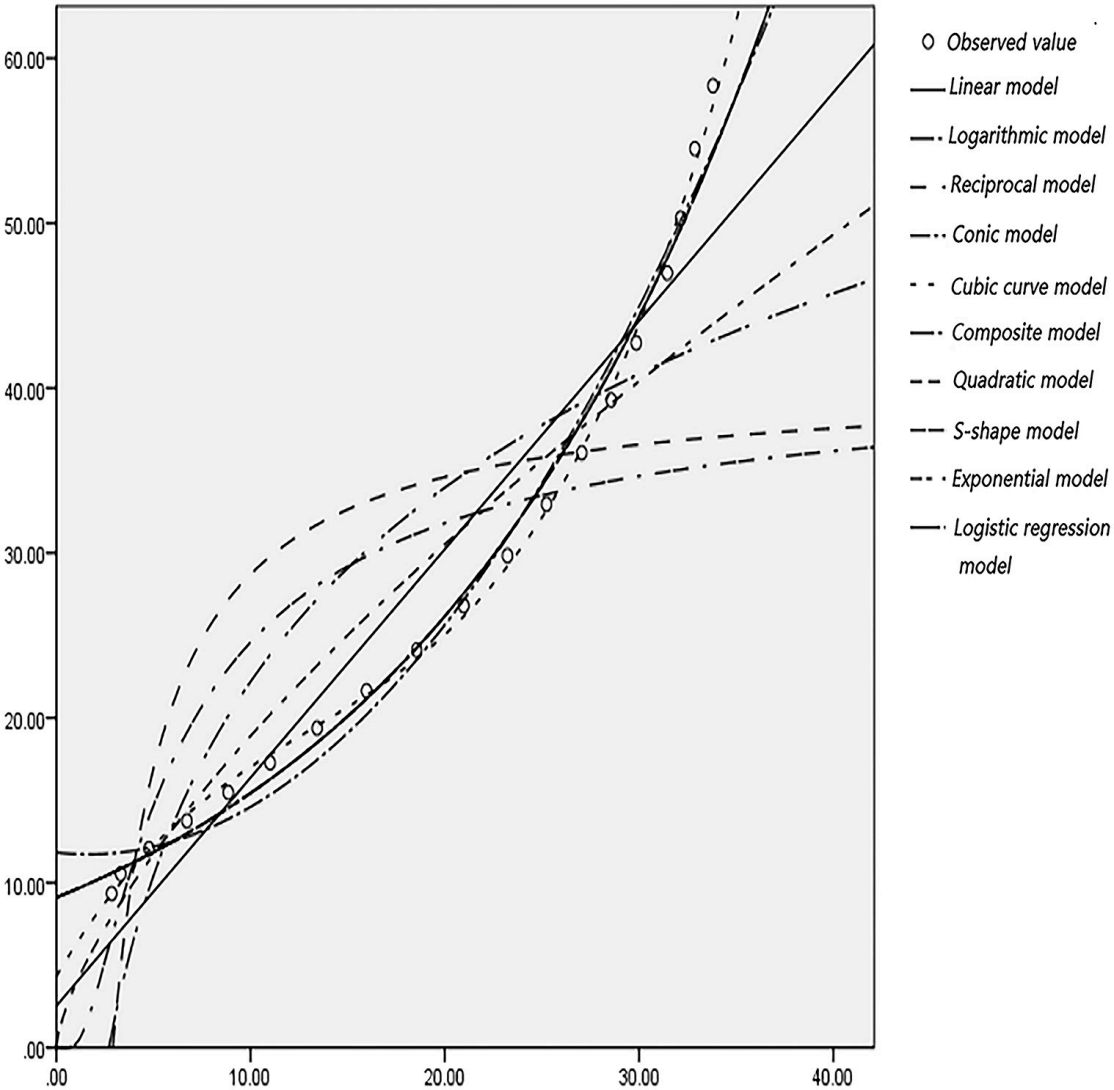
Curve fitting diagram of D1 at the intersection of Guizhong Avenue and Yingbin Road.

**Fig 11:**
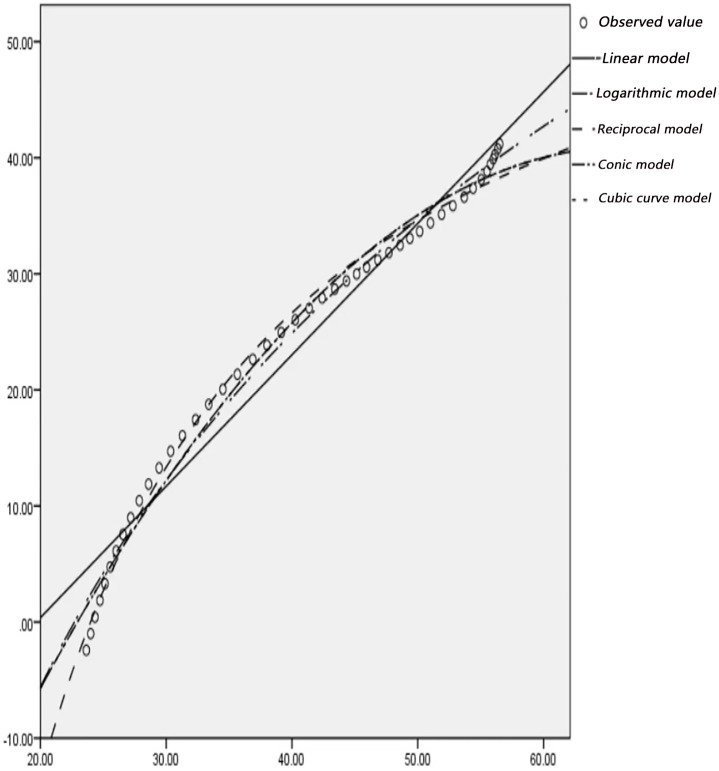
Curve fitting diagram of D2 at the intersection of Guizhong Avenue and Yingbin Road.

According to the actual observation of the trajectory point data of left-turning non-motorized vehicles, statistics and drawing analysis are carried out to determine the left-turning non-motorized vehicle expansion envelope D1, D2 at the intersection, where each circle represents a trajectory point. Use SPSS software to fit curves on D1 and D2, as shown in Figs 8–11. In order to represent the curves more accurately, comparing ten mathematical models, and the results are shown in Table 2.

**Table 2:** Curve estimation of outer envelope of left turn non-motor vehicles.

Intersection	Luban Road-Daxuedong Road		Guizhong Avenue-Yingbin Road	
Mathematical model	D1	D2	D1	D2
	*R* ^2^	*R* ^2^	*R* ^2^	*R* ^2^
*Linear model*	0.840	0.910	0.931	0.952
*Logarithmic model*	0.494	0.974	0.751	0.978
*Reciprocal model*	0.182	0.92875	0.489	0.987
*Conic model*	0.948	0.986	0.981	0.987
*Cubic curvemodel*	0.992	0.996	0.997	0.992
*Composite model*	0.958	0.757	0.991	-
*Quadratic model*	0.666	0.920	0.923	-
*S* − *shape model*	0.227	0.990	0.989	-
*Exponential model*	0.957	0.757	0.990	-
*Logistic regression model*	0.957	0.753	0.988	-

According to the data in Table 2, it can be seen that the fitting degree of D1, D2 and the cubic curve model is closest to 1, and the fitting effect is the best. To sum up, the cubic curve model of the expansion envelope D1 and D2 of the left-turning non-motorized vehicle is established as follows:

$$\begin{array}{ccc} \; & & y=\beta_{1}x^{3}+\beta_{2}x^{2}+\beta_{3}x+\beta_{0} \end{array}$$ (1)

In the formula, *y* is the ordinate of the track point of the non-motor vehicle; *x* is the abscissa of the track point of the non-motor vehicle; *β*_1_, *β*_2_, *β*_3_ are respectively coefficient; *β*_0_ is constant.

The cubic curve model fitting analysis is carried out on the expansion envelopes D1 and D2, and the parameter values in the model are obtained as shown in Tables 3 and 4:

**Table 3:** Coefficient of D1.

		*x*	*x* ^2^	*x* ^3^	Constant
Mathematical model	B	1.201	-0.049	0.001	13.734
	Standard error	0.236	0.011	0.000	1.198
Standardization *coefficient*	Beta	1.233	-2.669	2.478	-
*T*	-	5.094	-4.424	6.418	11.460
*Significance*	-	0.000	0.000	0.000	0.000

**Table 4:** Coefficient of D2.

		*x*	*x* ^2^	*x* ^3^	Constant
Mathematical model	B	1.191	-0.002	5.193 × 10^−^5	-11.331
	Standard error	0.142	0.003	0.000	1.824
Standardization *coefficient*	Beta	1.835	-0.289	-0.639	-6.211
*T*	-	8.386	-0.596	-2.295	-
*Significance*	-	0.000	0.057	0.031	0.000

The curve equation of the expansion envelope D1 can be obtained from Table 3

$$\begin{array}{ccc} \; & & y=0.001x^{3}-0.049x^{2}+1.201x+13.734 \end{array}$$ (2)

The curve equation of the expansion envelope D2 can be obtained from Table 4

$$\begin{array}{ccc} \; & & y=5.193\times 10^{-}5x^{3}-0.002x^{2}+1.191x-11.331 \end{array}$$ (3)

## 3.Expansion degree model of left-turning non-motor vehicles

In order to reflect the expansion effect of non-motor vehicle flow, the concept of expansion degree is introduced to describe the expansion phenomenon of non-motor vehicle flow. The width of the most apparent expansion in the passage space is defined as the maximum expansion width W, and the relationship between the maximum expansion width and the average width Db of the road occupied by bicycles after non-motorized vehicles start to expand is shown in Eq 4.

$$\begin{array}{ccc} \; & & D_{b}=W/N_{b} \end{array}$$ (4)

In the formula, *D*_*b*_ is the average lateral width of the road occupied by a single vehicle after non-motor vehicles start to expand, m; *W* is the maximum expansion width, m; *N*_*b*_ is the horizontal parallel number of left-turning non-motor vehicles when the expansion width is maximum, vehicles.

The expansion degree is the ratio of the lateral traffic flow density after non-motor vehicles start to expand and the lateral density of non-motor vehicles when they stand still in line.

$$\begin{array}{ccc} \; & & p^{k}=D_{b}/D_{0} \end{array}$$ (5)

In the formula, *p*^*k*^ is the expansion degree; *D*_0_ is the average lateral width of the road occupied by a single vehicle when the non-motorized vehicles stand in line, generally 0.8m.

**Fig 12:**
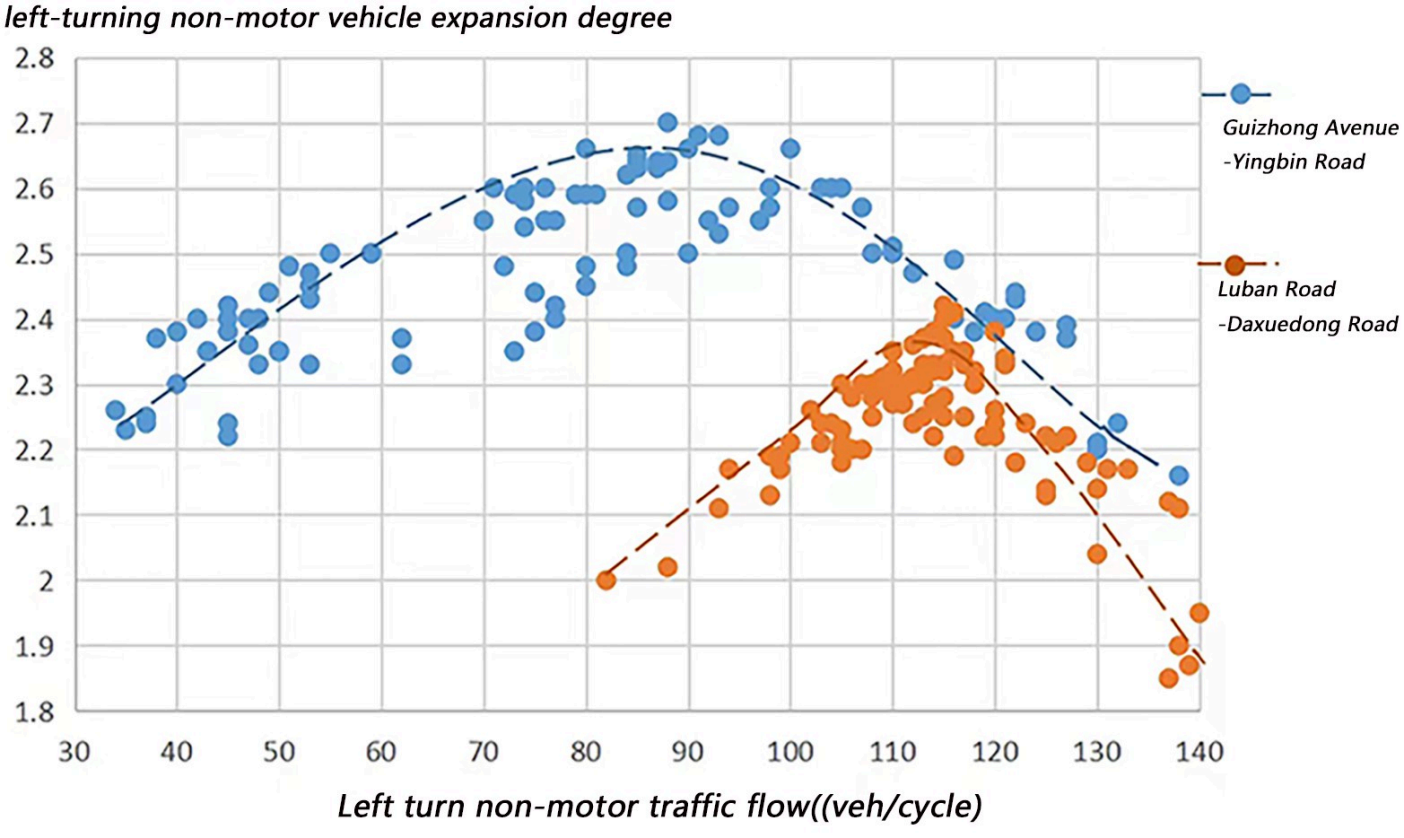
The relationship between expansion degree and left-turning non-motor vehicle flow.

**Fig 13:**
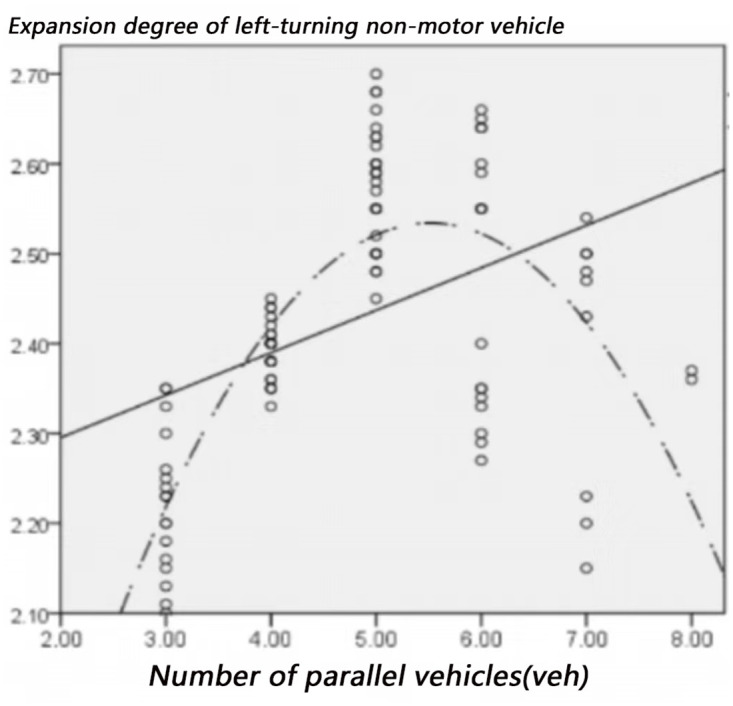
The relationship between expansion degree and the number of parallel non-motor vehicles.

**Fig 14:**
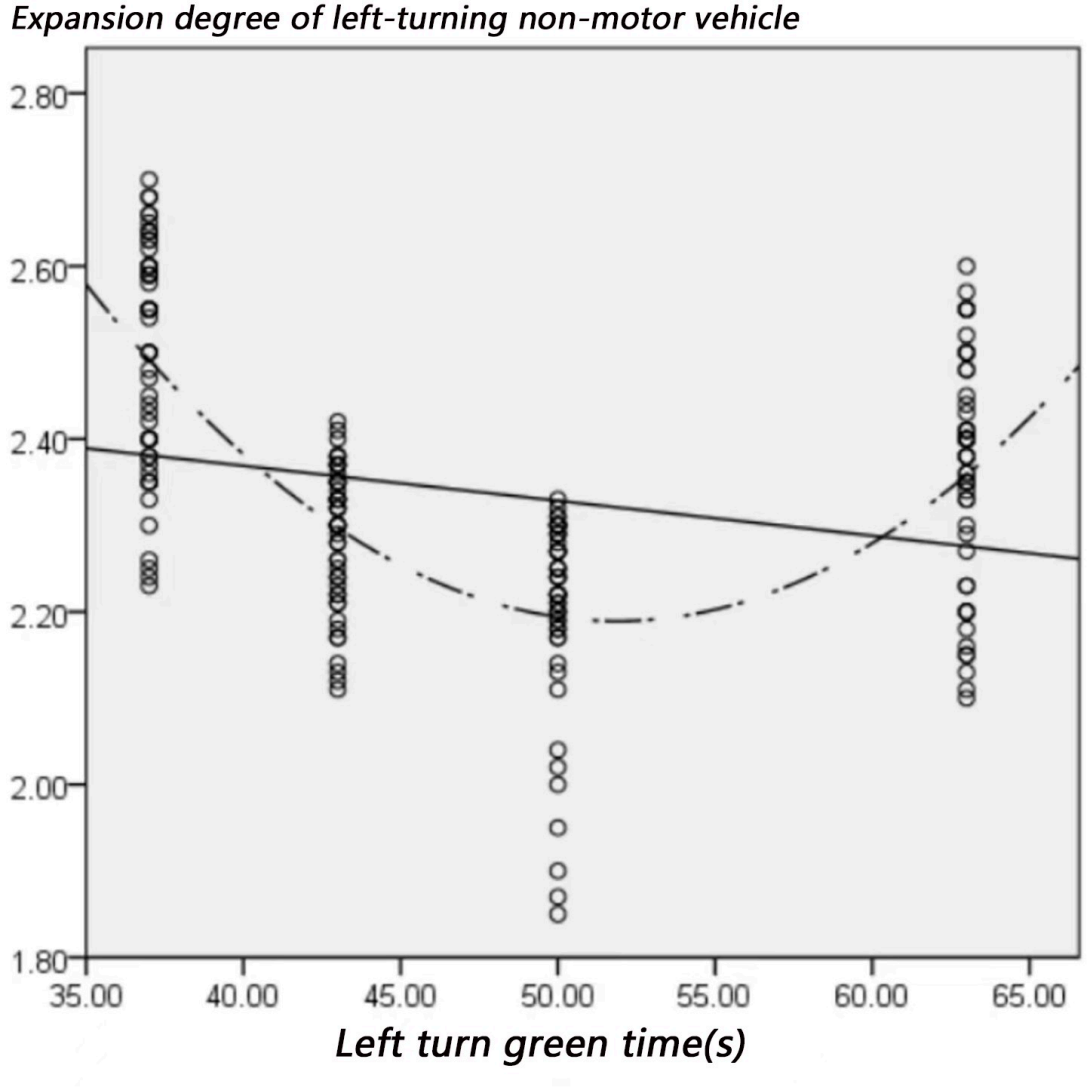
The relationship between expansion degree and green time of left turn.

The factors that may affect the expansion of left-turning non-motorized vehicles include: left-turning non-motorized vehicle flow, the number of parallel non-motorized vehicles, and the time of green light for left-turning. Figs 12–14 visually shows the relationship between the expansion degree of left-turning non-motorized vehicles and variables.

It can be found that the expansion degree presents a quadratic function curve trend with the increase of the variable value. Fig 12 shows that under the same non-motor vehicle flow conditions, the expansion degree of the intersection with a left-turn waiting area is smaller than that of an intersection without a left-turn waiting area. This is because of the setting of the left-turn waiting area, Left-turning motor vehicles occupy the original passage space of non-motor vehicles, and a large number of left-turning non-motor vehicles can only pass in a small space, resulting in a slight expansion, which affects the passage efficiency of non-motor vehicles. In the case of a small intersection space rather than a large motor vehicle flow, the orderly flow of non-motor vehicles will be guaranteed, and the traffic rate of motor vehicles will also increase accordingly.

For the intersection of Luban Road-University East Road, when the left-turn non-motor vehicle flow is less than 115, it increases with the increase of the left-turn non-motor vehicle flow, from 2.0 to 2.42, and when its flow is more significant than 115, the expansion degree starts With the increase of left-turning non-motor vehicle traffic, it gradually decreased to 1.85; the expansion degree of Guizhong Avenue-Yingbin Road intersection increased from 2.22 to 2.7, and when the non-motor vehicle traffic was less than 85, it decreased to 2.15.

Fig 13 shows that when the number of parallel non-motorized vehicles is small, the riding space between the non-motorized vehicles is sufficient, so the degree of expansion increases with the increase of left-turning motor vehicle traffic, while when the number of parallel non-motorized vehicles When it increases to a specific value, the expansion will not increase but decrease because the riding space of non-motor vehicles is limited due to factors such as the left-turn waiting area and motor vehicles turning left in the same direction.

Fig 14 shows that when the green light for non-motorized vehicles turning left is short, non-motorized vehicles tend to bypass the vehicle in front from the outside due to the cyclists’ eagerness to pass through the intersection, resulting in a larger overall traffic expansion width. As the green light time gradually increases, non-motorized vehicles have sufficient passing time so that the rear vehicles in the traffic flow will follow the front vehicles to pass through the intersection in an orderly manner so that the expansion width will show a downward trend. However, when the green light time exceeds a particular value, non-motor vehicles pass in a larger riding space, which increases the lateral diffusion of traffic flow, increasing the expansion width of left-turn non-motor vehicle flow. Nevertheless, when the green light time increases to a specific value, the number of non-motorized vehicles passing in a cycle is limited, so even if the green light time increases, the expansion width will not fluctuate significantly. In order to further verify the relationship between the influencing factors and the expansion degree of left-turning non-motorized vehicles, this paper conducts a Spearman correlation analysis on the left-turning non-motorized vehicle flow, the number of parallel non-motorized vehicles, and the time of green light for left-turning, as shown in Table 5.

**Table 5:** Spearman correlation analysis.

*Analysis index*	The correlation of Spearman’rho	Significance (bilateral)
*Expansion degree*	1	0.000
Left turn non-motor traffic flow	0.801	0.000
The number of parallel non-motorized vehicles	0.863	0.000
Left turn green time	0.602	0.000

Among them, the Spearman correlations of left-turn non-motor vehicle flow, parallel non-motor vehicle quantity, and left-turn green light time are 0.801, 0.863, and 0.602 respectively. The results show that the expansion degree has a high correlation with the left-turn non-motor vehicle flow and the number of parallel non-motor vehicles, while the Spearman correlation coefficient of the left-turn green light time is 0.602, which has a moderate correlation with the expansion degree. The expansion degree pk is affected by factors such as left-turn non-motor vehicle flow, parallel non-motor vehicle quantity, and left-turn green light time, and has a quadratic function relationship with variables, so the following relationship model is established.

$$\begin{array}{ccc} \; & & p^{k}=b_{0}+b_{1}Q_{1}^{2}+b_{2}Q_{1}+b_{3}N^{2}+b_{4}T^{2}+b_{5}N+b_{6}T \end{array}$$ (6)

In the formula, *b*_1_, *b*_2_, *b*_3_, *b*_4_, *b*_5_, *b*_6_ are the undetermined parameter, which is obtained according to the actual data fitting analysis; *b*_0_ is constant; *Q*_1_ is left turn non-motor vehicle flow, (vehicles/cycle); *N* is the number of parallel non-motor vehicles; *T* is green turn time (s).

According to the observation data, the nonlinear regression is carried out to obtain the fitted values of each parameter, as shown in Table 6.

**Table 6:** Estimated value of function parameters.

*Parameters*	*Estimation*	*Standard error*	Lower bound of 95% confidence interval	Upper 95% confidence interval
*b*_0_	0.060	0.561	-1.055	1.174
*b*_1_	0.000	0.000	0.000	0.000
*b*_2_	0.047	0.009	0.028	0.066
*b*_3_	-0.012	0.002	-0.016	-0.008
*b*_4_	0.000	0.000	0.000	0.000
*b*_5_	0.134	0.024	0.087	0.181
*b*_6_	-0.030	0.007	-0.044	-0.017

The fitting degree of the multiple nonlinear regression equation is *R*^2^ = 0.852, indicating that the nonlinear regression equation can better reflect the functional relationship between variables. The multiple nonlinear regression model of the left-turning non-motorized traffic expansion degree is shown in Eq (7).

$$\begin{array}{ccc} \; & & p^{k}=0.047Q_{1}-0.012N^{2}+0.134N-0.03T+0.06 \end{array}$$ (7)

## 4.Research on vehicle-bicycle conflicts caused by expansion characteristics

### 4.1 Conflict sample collection and processing

This paper collects conflict data between left-turning non-motor vehicles and left-turning motor vehicles. It takes whether motor vehicles and non-motor vehicles have the risk of collision as a qualitative criterion for machine-non-motor conflict discrimination.

Based on the principle of vehicle-bicycle conflicts generation, when the green light is on, non-motorized vehicles turning left start to accelerate quickly. Due to the characteristics of lateral expansion, when motor vehicles in the same direction pass through the intersection, some non-motorized vehicles encroach on motor vehicles in the same direction. Motor vehicles and some non-motor vehicles have horizontal conflicts.

Time to Collision (TTC) is a commonly used method for conflict discrimination. Internationally, 2.0s is used as the basis for judging conflicts. Domestic scholars use the 85% cumulative frequency method to obtain a conflict definition standard of TTC less than 1.6s. This paper uses 1.6s TTC as the criterion for judging non-machine conflicts, and the speed, position, acceleration, and other data of conflict samples are extracted, respectively, as shown in Table 7.

**Table 7:** Conflict sample data instance.

Serial number	Vehicle speed (m/s)	Non-motor vehicle speed (m/s)	Conflict distance(m)
1	2.90	4.40	2.5
2	1.30	3.80	4
3	2.50	5.0	3.0

According to statistical analysis, the intersection with left turn waiting area can significantly reduce the conflict between non-motor vehicles and motor vehicles. In this type of intersection, the motor vehicle can enter a special area to wait for the left turn, which will result in the motor vehicle running at a lower speed, thus reducing the conflict between the left turn non-motor vehicles and motor vehicles. On the contrary, the intersection without a left turn waiting area often occurs when non-motor vehicles accelerate to make a left turn in front of the motor vehicle after the green light starts. Due to the faster speed of non-motor vehicles, this situation will lead to more frequent conflicts between non-motor vehicles and vehicles.

### 4.2 Correlation test

This paper studies the number of conflicts between left-turning motor vehicles and left-turning non-motorized vehicles and finds that the most direct influencing factors are the flow of left-turning non-motorized vehicles, the number of parallel non-motorized vehicles and the expansion degree of left-turning non-motorized vehicles—factors to be examined in detail. Using SPSS software, the Spearman correlation test was carried out on the number of conflicts between left-turning motor vehicles and left-turning non-motorized vehicles and the influencing factors, and the results are shown in Table 8.

**Table 8:** Conflict sample data instance.

		TC	*Q*_1_	N	Expansion degree
TC	Spearman’s rho	1	0.870	0.727	0.602
	*Significance*	-	0.000	0.000	0.000
*Q*_1_	Spearman’s rho	0.870	1	-	-
	*Significance*	0.000	-	-	-
N	Spearman’s rho	0.727	1	-	-
	*Significance*	0.000	-	0.000	-
Expansion degree	*Spearman*′*srho*	0.602	-	1	-
	*Significance*	0.000	0.000	-	-

Through correlation analysis, it can be obtained that the simple correlation coefficient between the number of vehicle-bicycle conflicts at the intersection and each variable, the linear correlation coefficient between the left-turning non-motorized vehicle flow, the number of parallel non-motorized vehicles, the expansion degree of left-turning non-motorized vehicles and the number of vehicle-bicycle conflicts They are 0.870, 0727, and 0.602, respectively, and the significance is less than 0.01. Therefore, the multivariate linear regression equation of the number of vehicle-bicycle conflicts TC, the number of parallel non-motorized vehicles, and the expansion degree of left-turning non-motorized vehicles are established.

$$\begin{array}{ccc} \; & & TC=m_{0}+m_{1}Q_{1}+m_{2}N+m_{3}p^{k} \end{array}$$ (8)

In the formula, TC is the number of left turn vehicle-bicycle collisions (times/cycle); *m*_0_, *m*_1_, *m*_2_, *m*_3_ are regression coefficients.

Perform linear regression on the Model for the Number of vehicle-bicycle Conflicts to obtain the fitting values of each parameter, as shown in Table 9.

**Table 9:** Model coefficients for the number of vehicle-bicycle conflicts.

		Constant	Left turn non-motor traffic flow	Number of parallel non-motor vehicles	Expansion degree
Nonnormalized coefficient	B	-20.003	0.162	1.012	4.210
	Standard error	3.690	0.010	0.096	0.010
Standardization coefficient	Beta		0.658	0.597	0.164
T		-5.421	15.995	10.563	3.028
Significance		0.000	0.000	0.000	0.000

Theoretically, when the flow of left-turning non-motorized vehicles, the expansion width of left-turning non-motorized vehicles, and the number of parallel non-motorized vehicles are all 0, the number of vehicle-bicycle Conflicts is 0, so under the premise of *TC* > 0, The calculation model is shown in Eq (9). The goodness of fit test was performed on the number of vehicle-bicycle conflicts model, as shown in Tables 10 and 11.

$$\begin{array}{ccc} \; & & TC=0.162Q_{1}+1.012N+4.210p^{k}-20.003 \end{array}$$ (9)

**Table 10:** Goodness of fit test table of the number of vehicle-bicycle conflicts.

Model	1
R	0.968
*R* ^2^	0.936
*AdjustedR* ^2^	0.932
*Standardskewerror*	0.78679

**Table 11:** *ANOVA*^a^.

Model		Sum of squares	df	Mean squared	F	Significance
1	Regression	419.304	3	139.768	225.784	0.000^b^
	Residual	28.476	46	0.619	-	-
	Total	447.780	49	-	-	-

^a^. Dependent variables: Number of conflicts.

^b^. Predicted value: (constant), Left turn non motor traffic flow, Number of parallel vehicles, Expansion degree.

The goodness of fit test and variance analysis was carried out on the the number of vehicle-bicycle conflicts model. The *R*^2^ of the regression function was 0.932, indicating that the model could better reflect the relationship between variables. *p* < 0.001 indicating that the equation has passed the significance test and the linear relationship of the equation is proven to be significant. According to the analysis of variance, F of the model is 225.784.

### 4.3 Model verification

In order to verify the accuracy of the number of vehicle-bicycle conflicts model, another 36 sets of test sample data were counted, each variable parameter was brought into the model formula, and the left-turn machine non-conflict number was calculated, and the calculated value was compared with the actual measurement Values are compared to judge the degree of relative error between them, as shown in Table 12.

**Table 12:** Example of comparison results of vehicle-bicycle collision number models.

sample	Left turn non-motor traffic flow (veh/cycle)	Number of parallel vehicles (veh)	Expansion degree	Computed collision number	Actual conflict number	Absolute value of error	Relative value of error
1	120	8	2.20	16.795	16	0.795	4.97%
2	95	5	2.35	10.3405	10	0.3405	3.41%
3	112	7	2.22	14.5712	14	0.5712	4.08%
4	138	10	1.98	20.8088	19	1.8088	9.52%
5	106	6	2.30	12.924	12	0.924	7.70%
6	115	8	2.20	15.985	15	0.985	6.57%
7	125	9	2.18	18.5328	17	1.5328	9.02%
8	135	10	1.92	20.0792	19	1.0702	5.63%
9	143	10	1.85	21.0715	20	1.0715	5.36%
10	88	5	2.4	9.417	8	1.417	17.71%
11	92	5	2.36	9.8966	9	0.8966	9.96%
12	120	8	2.2	16.795	16	0.795	4.97%
13	139	10	1.86	20.4656	20	0.4656	2.33%
14	142	9	1.9	20.108	20	0.108	0.54%
15	97	6	2.32	11.5502	11	0.5502	5.00%
16	99	6	2.33	11.9163	11	0.9163	8.33%
17	104	6	2.28	12.5158	12	0.5158	4.30%
18	109	7	2.25	14.2115	14	0.2115	1.51%
19	116	8	2.21	16.1891	16	0.1891	1.18%
20	132	10	1.95	19.7105	19	0.7105	3.74%
21	130	9	2.02	18.6692	20	1.3308	6.65%
22	111	8	2.2	15.337	17	1.663	9.78%
23	95	6	2.36	11.3946	12	0.6064	5.05%
24	85	5	2.4	8.931	9	0.069	0.77%
25	106	6	2.32	13.0082	12	1.0082	8.40%
26	113	7	2.27	14.9437	16	1.0563	6.60%
27	121	8	2.23	17.0833	16	1.0833	6.77%
28	115	8	2.17	15.8587	15	0.8517	5.72%
29	108	7	2.31	14.3021	16	1.6979	10.61%
30	117	8	2.24	16.4774	15	1.4774	9.85%
31	122	8	2.25	17.3295	19	1.6705	8.79%
32	126	9	2.2	18.779	18	0.779	4.33%
33	118	9	1.98	16.5568	15	1.5568	10.38%
34	104	6	2.31	12.6421	12	0.6421	5.35%
35	94	6	2.35	11.1905	12	0.8095	6.75%
36	132	9	2	18.909	20	1.091	5.46%

According to the comparison of the calculation results of the number of vehicle-bicycle conflicts model and the actual survey statistics of 36 sets of data, we can draw a conclusion: the relative error value of the model is 6.3077%, which can verify that the model has high accuracy.

## 5.Conclusion

- Select two intersections in Nanning City and Laibin City, Guangxi, use video trajectory tracking technology to extract the trajectory data of non-motor vehicles turning left to cross the street for analysis, and use SPSS software to perform curve fitting on the expansion envelopes D1 and D2, establish the cubic curve model of the expansion envelope with the highest fitting degree, the fitting coefficient *R*^2^ is more significant than 0.990, and the fitting effect is the best.
- According to statistical analysis, the variation range of the expansion degree of the intersection without a left-turn waiting area is 2.10~2.70, and the expansion degree of the intersection with a left-turn waiting area is 2.0~2.42. The left-turning non-motorized vehicle flow, the number of parallel non-motorized vehicles, the time of the left-turning green light, and the expansion degree of left-turning non-motorized vehicles have a strong correlation, and the expansion degree and its influencing factors present an apparent quadratic function trend. The fitting effect is high for the multiple nonlinear regression model of expansion degree and the verification model fitting degree *R*^2^ = 0.852.
- According to the determined vehicle-bicycle conflicts threshold, establish a model of left-turning non-motorized vehicle flow, expansion degree, and the number of parallel non-motorized vehicles to the number of left-turning non-conflicted vehicles. After 36 sets of test data verification, the relative error value is 6.3077%, the model has higher accuracy.

## 6.Limitations and future work

There are some important limitations in this paper, which can be divided into the following three aspects:

1. Insufficient data collection: The research on the expansion characteristics of non-motor vehicles requires a large amount of data support, but the actual data collection is still small, and the data collection under different traffic environments is insufficient, resulting in the reliability and universality of the research results to be improved.
2. Single object of study: At present, the research on the expansion characteristics of non-motor vehicles mainly focuses on bicycles, and there are relatively few studies on the expansion characteristics of other non-motor vehicles, so it is necessary to expand the scope of research objects in the future.
3. Insufficient research depth: Current research on the expansion characteristics of non-motor vehicles mainly focuses on local traffic environments such as non-motor lanes and intersections, and further research is needed on the impact of the expansion characteristics of non-motor vehicles on urban traffic networks and the overall efficiency of urban traffic.

In the future, the influence of non-motor vehicle driver behavior characteristics and traffic environment on the expansion characteristics of non-motor vehicles can be deeply discussed, and an intersection signal control method based on the expansion characteristics of non-motor vehicles can be proposed. The intersection signal control parameters can be flexibly adjusted according to the differences in the expansion characteristics of non-motor vehicles of different types and speeds, to improve the traffic efficiency and safety of the intersection. In addition, the influence of the expansion characteristics of non-motor vehicles on the width and number of non-motor vehicle lanes and the length and width of the left bend waiting area can also be explored, and reasonable Settings can be made according to the actual situation to meet the demand of non-motor vehicle flow, reduce the traffic safety hazards caused by the expansion characteristics, and formulate a more scientific and reasonable urban traffic planning and design scheme.

## Supporting information

S1 File It contains all the data files for this manuscript. (XLSX)
